# Local structure supports learning of deterministic behavior in recurrent neural networks

**DOI:** 10.1186/1471-2202-16-S1-P195

**Published:** 2015-12-18

**Authors:** Jonathan Binas, Giacomo Indiveri, Michael Pfeiffer

**Affiliations:** 1 Institute of Neuroinformatics, University of Zurich, Zurich, Switzerland; 2 ETH Zurich, Zurich, Switzerland

## 

Many aspects of behavior, such as language, navigation, or logical reasoning require strongly deterministic and sequential processing of sensory and internal signals. This type of computation can be modeled conveniently in the framework of finite automata.

In this study, we present a recurrent neural network based on biologically plausible circuit motifs, which is able to learn such deterministic behavior from sensory input and reinforcement signals. We find that simple, biologically plausible structural constraints lead to optimized solutions and significantly improve the training process.

**Figure 1:**
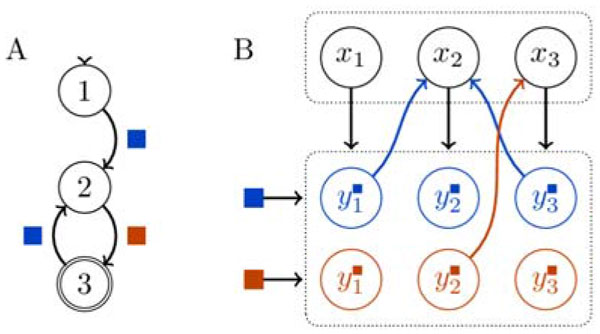
**Abstract graphical representation of an example finite automaton (A) and corresponding neural implementation (B)**. The dotted boxes represent Winner-Take-All circuits in which only one population is active at a time. The populations labeled *x *represent the states while the populations labeled *y *implement the conditional transitions between states. The colored connections are learned during training.

Previous work [1,2] has shown how arbitrary finite automata can be hand-crafted in simple networks of neural populations by interconnecting multiple Winner-Take-All units - small circuit motifs that match the properties of cortical canonical microcircuits [3,4]. Figure 1 illustrates this transformation from an automaton to neural network with populations of neurons encoding either the state or potential state transitions. We extend that work by introducing a reinforcement learning mechanism whose weight updates take the form of reward-modulated Hebbian rule. This mechanism leads to reconfiguration of the network connectivity in such a way that a desired behavior is learned from sequences of inputs and reward signals.

As a key result of our study, we find that simple constraints on the network topology, favoring local connectivity patterns, lead to dramatic improvements both in training time and in the optimality of the found solution, where the optimum is defined as the automaton with the minimum number of states used to implement a given behavior. These structural constraints correspond well to biological neural systems, where short-range connections far outnumber long-range ones.
